# Microstructural Alterations in Hippocampal Subfields Mediate Age-Related Memory Decline in Humans

**DOI:** 10.3389/fnagi.2020.00094

**Published:** 2020-04-09

**Authors:** Hamsanandini Radhakrishnan, Shauna M. Stark, Craig E. L. Stark

**Affiliations:** ^1^Mathematical, Computational and Systems Biology, University of California, Irvine, Irvine, CA, United States; ^2^Department of Neurobiology and Behavior, University of California, Irvine, Irvine, CA, United States

**Keywords:** hippocampus, diffusion-weighted imaging, NODDI, fornix, aging

## Abstract

Aging, even in the absence of clear pathology of dementia, is associated with cognitive decline. Neuroimaging, especially diffusion-weighted imaging, has been highly valuable in understanding some of these changes in live humans, non-invasively. Traditional tensor techniques have revealed that the integrity of the fornix and other white matter tracts significantly deteriorates with age, and that this deterioration is highly correlated with worsening cognitive performance. However, traditional tensor techniques are still not specific enough to indict explicit microstructural features that may be responsible for age-related cognitive decline and cannot be used to effectively study gray matter properties. Here, we sought to determine whether recent advances in diffusion-weighted imaging, including Neurite Orientation Dispersion and Density Imaging (NODDI) and Constrained Spherical Deconvolution, would provide more sensitive measures of age-related changes in the microstructure of the medial temporal lobe. We evaluated these measures in a group of young (ages 20–38 years old) and older (ages 59–84 years old) adults and assessed their relationships with performance on tests of cognition. We found that the fiber density (FD) of the fornix and the neurite density index (NDI) of the fornix, hippocampal subfields (DG/CA3, CA1, and subiculum), and parahippocampal cortex, varied as a function of age in a cross-sectional cohort. Moreover, in the fornix, DG/CA3, and CA1, these changes correlated with memory performance on the Rey Auditory Verbal Learning Test (RAVLT), even after regressing out the effect of age, suggesting that they were capturing neurobiological properties directly related to performance in this task. These measures provide more details regarding age-related neurobiological properties. For example, a change in fiber density could mean a reduction in axonal packing density or myelination, and the increase in NDI observed might be explained by changes in dendritic complexity or even sprouting. These results provide a far more comprehensive view than previously determined on the possible system-wide processes that may be occurring because of healthy aging and demonstrate that advanced diffusion-weighted imaging is evolving into a powerful tool to study more than just white matter properties.

## Introduction

Decades of research have shown that, even outside of overt pathology or dementia, aging is associated with cognitive decline, such as decreases in processing speed, poorer divided attention, and episodic memory impairments (Johnson, 1997; Schacter et al., 1997; Glisky, 2007; Eckert, 2011). While there are a host of changes in the brain that have been tied to age-related cognitive decline, structural and functional alterations in the hippocampus and other regions of the medial temporal lobe likely mediate much of these alterations of memory (Morrison and Baxter, 2012; Stark and Stark, 2017b). Human imaging studies have shown that the hippocampal volume decreases after the age of 70 at a rate of approximately 1.5% a year (Jack et al., 1998; Raz et al., 2005). This reduction could be due to synaptic size reduction (Petralia et al., 2014), microglia decrease (Sharaf et al., 2013), demyelination (Peters, 2002; Kövari et al., 2004) and/or other changes in connectivity (Fjell et al., 2016). More subtle changes are also observed in individual neurons, such as shrinkage in soma size (Ahmad and Spear, 1993) and a reduction or regression in dendritic branching (Scheibel et al., 1975). Aging also results in axonal degeneration of the fornix and other white matter pathways, due to loss of myelinated fibers and alterations in the myelin sheath (Peters et al., 2010; Salvadores et al., 2017). Studies of these underlying neurobiological changes associated with age have largely been performed in animal models as direct studies in humans are often infeasible. However, new neuroimaging techniques may prove to be valuable tools for investigating these age-related alterations *in vivo* in the human brain.

Diffusion tensor imaging (DTI) has enabled us to probe white matter changes using measures like fractional anisotropy (FA) and mean diffusivity (MD), providing some form of *in vivo* measure of microstructure and integrity (Table 1). In the fornix, FA is reduced and MD is increased as a consequence of aging, reflecting a reduction in white matter integrity (Gunning-Dixon et al., 2009; Kochunov et al., 2012; Madden et al., 2012; Bennett and Madden, 2014; Bennett and Stark, 2015). These measures are also correlated with cognitive performance in both humans and rodents (Takahashi et al., 2000; Charlton et al., 2007; Kantarci, 2014). Though DTI has been extremely useful for studying microarchitectural properties in white matter and its influence on behavior, it is inherently a nonspecific technique. A change in FA could be caused by changes in myelination, axon diameter, membrane permeability, or axon packing density (Sampaio-Baptista and Johansen-Berg, 2017). DTI is also incapable of capturing complex microstructural details within a given voxel, which is particularly important in regions of crossing, kissing, and fanning fibers (Zhang et al., 2012; Jeurissen et al., 2013).

**Table 1:** Summary of metrics used in this study.

Metric	Abbreviation	Description	Range
Fractional anisotropy	FA	A measure of axonal organization or integrity based on the coherence of orientations of the bundles. Mainly used to study white matter, and generally decreases with age. Reductions in FA can mean neurodegeneration, a myelin sheath depletion or just general atrophy of fiber bundles (Song et al., 2003; Budde et al., 2007).	0 (most isotropic) −1 (least isotropic)
Mean diffusivity	MD	Another measure of white matter bundle integrity calculated as the average amount of water diffusion inside the voxel. MD in most regions increases with age, also suggesting demyelination or axonal degradation (Abe et al., 2002; Grieve et al., 2007; Hsu et al., 2008).	Continuous (directly proportional to the amount of diffusion).
Fiber Density	FD	Calculated as the integral of a given fixel’s FOD. Directly proportional to the intra axonal volume of the fiber population aligned with the given fixel (Raffelt et al., 2012).	0 (Least dense) −1 (Most dense)
Fiber cross section	FC	Captures individual differences in the diameters of distinct fiber bundles. Computed as the amount of distortion necessary to warp a given FOD to the same FOD in template space (Raffelt et al., 2017).	0 (least diameter) −1 (most diameter)
Fiber density and cross section	FDC	A joint metric of FD and FC calculated as their product. Captures both microstructural properties as well as more large-scale changes within bundles.	0–1
Neurite density index	NDI	Calculated as the proportion of the voxel expressing unhindered diffusion along a given set of sticks, and also restricted diffusion perpendicular to the same set of sticks. Might be able to pick up on the number of neurites or the complexity of their dendrites (Billiet et al., 2015).	0 (most extracellular) −1 (most intracellular)
Orientation dispersion index	ODI	The measure of tortuosity coupling an intracellular and extracellular space. Gives the variability of neurite orientations, and might be able to pick up on the dispersion of axons and neurons within a voxel (Billiet et al., 2015).	0 (Least dispersed) −1 (Most dispersed)
Fractional isotropy	FISO	The measure of the amount of isotropic free volume within a voxel- and is usually proportional to the amount of cerebrospinal fluid in a voxel. Might also pick up on other free water entities like dead cells (Billiet et al., 2015).	0 (Least CSF) −1 (Most CSF)

Recent advances in diffusion imaging like multiple tensor models to Q-Ball and Q-space imaging (King et al., 1994; Tuch, 2004; Tournier et al., 2004) have attempted to address problems of complex fiber architecture, but these methods are still not fiber specific or easily assignable to segmented white matter pathways. Moreover, as we will show, voxel-based analysis of these metrics may yield false-positive differences between groups as multiple pathways can pass through a voxel, further confounding how we interpret “pathway-specific” metrics. To address this issue, fixel-based analysis (Raffelt et al., 2017) is one of the first techniques that enable tract-specific statistical analysis. Here, a “fixel” refers to a particular fiber population inside a voxel (Raffelt et al., 2015). Using constrained spherical deconvolution, this method can estimate the total intra-axonal volume of white matter axons in any direction, enabling the detection of tract-specific degeneration. This technique can estimate microstructural changes (fiber density), macrostructural changes (fiber cross-section), and the differences arising from a combination of both classes of degeneration (see Table 1; Raffelt et al., 2017). These metrics have proven to be more sensitive to microarchitectural alterations and more useful in revealing minute but clinically relevant disease-associated differences, as compared to traditional tensor-based analysis (Mito et al., 2018). However, very few studies have explored such changes associated with healthy aging and none (to our knowledge) have looked at age-related fixel-based decline in the fornix and its impact on cognitive performance.

In addition to problems with specificity, DTI is also not suited for studying gray matter architecture, as the complexity in cell layers cannot be detected by simple tensor approaches. Neurite Orientation Dispersion and Density Imaging (NODDI; Zhang et al., 2012) addresses this problem using multi-compartment diffusion modeling, in which restricted diffusion is modeled as a set of sticks, hindered diffusion as the dispersion of the sticks, and unrestricted diffusion as an isotropic sphere. These metrics are not only completely agnostic to tissue type (all voxels are modeled by the same set of equations), but also provide a more comprehensive analysis of the microstructural subtleties and underlying mechanisms associated with disease or development-induced changes. NODDI has been extensively used to study both pathological and normal brain development (Adluru et al., 2014; Kunz et al., 2014; Eaton-Rosen et al., 2015; Jelescu et al., 2015; Wen et al., 2015; Grussu et al., 2017), and many recent studies have explored how healthy aging can influence these metrics, shedding some light on their potential biological implications. For example, the orientation dispersion index (ODI) has been shown to decrease globally in human gray matter with age, suggesting a reduction in dendrite complexity or arborization (Nazeri et al., 2015). There have also been reports of increased neurite density index (NDI) and ODI in localized white matter regions, primarily in the frontal lobe (Billiet et al., 2015; Chang et al., 2015).

Given these advantages in NODDI, this study aims to focus on aging-induced changes in neurite density, dispersion, and fiber population metrics in the medial temporal lobe and their relationship with cognitive performance. Venkatesh et al. (submitted) have recently shown that neurite density, dispersion and free water volume concentration all increase with age in the human hippocampus, and have shown that NODDI metrics are better at predicting age than traditional diffusion tensor measures. However, there have been no reports exploring age-related changes in NODDI metrics in hippocampal subfields, or how they relate to changes in memory performance. Here, we sought to determine the effect of age on both fiber metrics, as well as NODDI properties, in the medial temporal lobe. We further assessed the relationship between changes in these metrics and cognitive performance in young and older adults. Finally, we used structural equation modeling to assess the extent to which these structural changes drive age-related cognitive decline.

## Materials and Methods

### Participants

Forty-eight adults were recruited from the Orange County area in California. Three subjects were excluded for data segmentation issues, three subjects were excluded for registration issues, and four were excluded for neuropsychological scores more than two standard deviations below the mean for their age group. The final study adults consisted of 15 young (20–38 years, 28.4 ± 4.6 years, eight females) and 23 older (59–84 years, 69.9 ± 5.3 years, 14 females) adults. All participants provided informed consent before participation in this study, approved by the University of California, Irvine Institutional Review Board, and were compensated for their time.

### Neuropsychological Battery

All participants completed a battery of neuropsychological tests to evaluate their cognitive abilities. Tests included the Mini-mental State Examination (MMSE) to screen for cognitive impairment (Folstein et al., 1975), Rey Auditory Verbal Learning Test (RAVLT) to evaluate memory recall and recognition (Rey, 1941), Geriatric Depression Scale (GDS) and Beck Depression Index (BDI) to characterize their depression profiles (Beck, 1972; Yesavage et al., 1982; no participant was found to have a profile in the moderate-to-severe range), Trails A and B, Stroop, and Letter Number Sequencing to assess executive functioning (Stroop, 1935; Wechsler, 1997a; Reitan and Wolfson, 1985), and Digit Span to characterize working memory (Wechsler, 1997b; see Table 2). The Mini-Mental State Exam (MMSE) total score is the sum of all test questions (maximum score of 30). The RAVLT has three components: five presentations of the same 15-word lists with immediate recall, a second immediate recall test following an interference list of 15 novel words, and a final recall after a 15-min delay. Here, the RAVLT Delay reflects the final recall score (maximum score of 15). The Rey-Osterrieth Complex Figure score has two components: Rey-O Figure reflects the number of correct components drawn from memory after a 15-min delay when the figure is no longer present (maximum score of 38). We report the total time in seconds to complete Trails A and B. The Stroop Color-Word reflects the number of words read aloud correctly in 1 min when the color of ink does not match the word (e.g., the word “red” in green ink). The Letter Number Sequencing score reflects the number of correct sequences of letters and numbers recalled immediately after hearing them (e.g., the correct response to “A-6-H-3” would be “A-H-3–6”; maximum score of 21). Similarly, the total Digit Span score reflects the number of correct digits recalled, both forward and backward versions (maximum score of 30).

**Table 2:** Demographics and neuropsychological test scores.

Demographics	Young	Older	*T*-stat	*p*-value
N	15	23		
Mean age	28.40 ± 4.73	69.87 ± 5.43		
Education	17.00 ± 2.17	17.30 ± 1.77		
MMSE	29.40 ± 0.74	29.48 ± 0.67	−0.33	0.37
RAVLT total	**61.13 ± 5.05**	**55.00 ± 8.06**	**2.88**	**0.003**
RAVLT immediate	**13.80 ± 1.42**	**12.21 ± 2.50**	**2.48**	**0.009**
RAVLT delay	**13.86 ± 1.18**	**12.00 ± 2.71**	**2.9**	**0.003**
Trails A	**16.27 ± 5.86**	**24.52 ± 7.79**	**−3.72**	**0.0004**
Trails B	**45.40 ± 13.43**	**66.39 ± 21.06**	**−3.75**	**0.0003**
Stroop Color-Word	**54.27 ± 6.75**	**48.21 ± 5.45**	**2.1**	**0.02**
Digit Span	**21.47 ± 4.27**	**18.65 ± 3.64**	**2.91**	**0.004**
Rey-O Figure	35.4 ± 1.12	34.6 ± 2.79	1.22	0.11
Rey-O Delayed	**25.00 ± 5.96**	**16.85 ± 6.37**	**4.01**	**0.0002**

*Demographics and neuropsychological test scores. Entries in bold indicate properties that have significant group differences (*p* < 0.05)*.

### MR Image Acquisition

The participants were scanned using a Philips Achieva 3.0 Tesla MRI system, using a 32-channel SENSE receive-only head coil. Fitted padding was used to minimize head movements. A T1-weighted magnetization-prepared rapid gradient echo (MP-RAGE) scan was acquired (TR = 11 ms, TE = 4.6 ms, flip angle = −18°, 200 sagittal slices and 0.75 mm isotropic resolution) for structural analysis and registration. High-resolution structural MRI images of the MTL were acquired using a T2-weighted sequence to aid in MTL segmentation (TE = 80 ms, flip angle = 90°, slices = 54, slice thickness = 3 mm, matrix size = 384 × 384, voxel size = 0.469 × 0.469 × 2 mm, and an in-plane field of view = 108 × 180 mm). Both structural images were aligned as oblique coronals perpendicular to the long axis of the hippocampus and positioned to ensure MTL coverage. Three diffusion-weighted scans (TR = 2,174–2,734 ms, TE = 94 ms, 80 axial slices and 1.69 mm isotropic resolution) were acquired for four gradient values: *b* = 500, 1,000, 2,000, and 2,500 s/mm^2^. Gradients were applied in 10 directions for each scan (120 directions in total) along with 12 images with no diffusion weighting (*b* = 0).

### Diffusion Data Preprocessing

**Figure 1:**
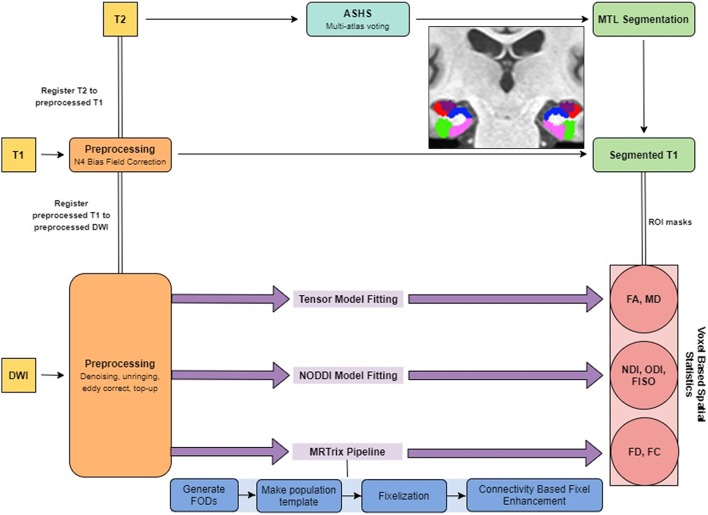
Summary of the analysis pipeline.

All preprocessing steps employed MRtrix3^1^ commands or used MRtrix3 scripts that linked external software packages. Physiological noise arising from the thermal motion of water molecules in the brain was first removed (Veraart et al., 2016), followed by removal of Gibbs ringing artifacts (Kellner et al., 2016), eddy current correction (Andersson and Sotiropoulos, 2016) and bias field correction (Tustison et al., 2014). The image intensity was then normalized across subjects in the log-domain (Raffelt et al., 2012; Figure 1).

### Structural Data Preprocessing

The T1w images were corrected for intensity inhomogeneities using Advanced Normalization Tools (ANTs) N4 bias correction. Each individual’s structural image was then nonlinearly registered to their respective preprocessed b0 image so that the structural and diffusion images were in the same space for the rest of the analyses. Registration was manually checked to ensure accuracy. To segment the MTL, we used a multi-atlas model created by our lab using ASHS (Yushkevich et al., 2010) and 19 independent hand-segmented brains (both the MP-RAGE and high-resolution T2 images). These segmentations included both segmentations of the parahippocampal gyrus into perirhinal (PRC), parahippocampal (PHC), and entorhinal (ERC) cortices described previously (Insausti et al., 1998; Stark and Okado, 2003). Similarly, we segmented the hippocampus into three subregions: a combined dentate gyrus and CA3 (combined due to resolution constraints; DG/CA3), CA1, and subiculum, based on our previous work (Stark and Stark, 2017a). For each of these, we created multi-atlas models in ASHS and then used these to segment each individual’s high-resolution T2 scan.

### Fiber Orientation Distribution Analysis With MRtrix3

Following preprocessing, we generated response functions for white matter, gray matter, and CSF for each participant. The response function for each tissue type was then averaged across subjects. The fiber orientation distributions (FODs) were then calculated for each tissue type from the group averaged response functions using Multi-Shell Multi-Tissue Constrained Spherical Deconvolution (MSMT-CSD; Dhollander et al., 2016). We created a study-specific template using an iterative registration and averaging approach (Raffelt et al., 2011) using the white matter FODs from 20 arbitrary subjects (10 old and 10 young). All the subjects’ FODs were registered to this template using a FOD-guided non-linear registration (Raffelt et al., 2011). The remaining analysis in MRtrix3 was performed in this study-specific template space, unless mentioned otherwise.

To segment the white matter tracts in a common space, we generated a tractogram from the template using whole-brain probabilistic tractography (20 million streamlines, termination cutoff = 0.6). To account for reconstruction biases, we filtered the tractogram to 2 million streamlines using the Spherical-deconvolution Informed Filtering of Tractograms algorithm (Smith et al., 2013).

The FOD images were then segmented into “fixels” (individual voxels sectioned into individual fibers) for further analysis. We calculated the fiber density (FD), and the fiber bundle cross-section (FC) for each subject across all white matter fixels (Raffelt et al., 2017). The FD of a given fixel is proportional to the intra-axonal volume of axons aligned in a given direction and is calculated as the integral of the FOD along that direction, using the Apparent Fiber Density framework (Raffelt et al., 2012). The FC metric is meant to capture macrostructural changes and is sensitive to axonal loss and pathway atrophy (Grazioplene et al., 2018; Pannek et al., 2018). It is calculated as the amount of distortion perpendicular to a given fixel’s orientation that is required to warp the individual’s FOD to the template FOD (Raffelt et al., 2017). We used the natural logarithm of this metric for statistical analysis to ensure that the data were normally distributed and centered around zero. For comparison purposes, we also computed traditional fractional anisotropy (FA) and mean diffusivity (MD) metrics.

The fixel metrics were compared across age groups at each white matter fixel using a General Linear Model. We performed connectivity-based smoothing and statistical inference using connectivity-based fixel enhancement (CFE). Family-wise error-corrected *p*-values were then assigned to each fixel using non-parametric permutation testing of the CFE enhanced *t-statistics* (Nichols and Holmes, 2002).

### NODDI Analysis With the Microstructure Diffusion Toolbox (MDT)

Microstructure metrics were calculated using the Neurite Orientation Dispersion and Density Imaging (NODDI) model (Zhang et al., 2012) with the Microstructure Diffusion Toolbox (MDT; Harms et al., 2017). NODDI characterizes diffusion within each brain voxel as a combination of intracellular, extracellular and CSF based components. The intracellular compartment seeks to capture neurite membranes and myelin sheaths and is modeled as a set of sticks with restricted diffusion perpendicular to the orientation of the axonal bundles and unhindered diffusion along them. The extracellular compartment is thought to capture primarily the space around the neurites, composed of glial cells and somas. The diffusion in this space is modeled as hindered Gaussian anisotropic diffusion. Finally, the CSF compartment is modeled as isotropic diffusion. The NDI gives the fraction of tissue volume restricted within neurites. It scales from 0 to 1, with 0 being most extracellular-like diffusion and 1 being most intracellular-like (Billiet et al., 2015). The ODI is a measure of tortuosity and is calculated as the dispersion coefficient of the neurites. An ODI closer to 0 is indicative of well-aligned neurites, while that closer to one indicates higher levels of dispersion. The fractional isotropy (FISO) is the percentage of the volume in each voxel that is best modeled by free-water diffusion. The 4D DWI data was passed in as input and parametric maps of NDI, ODI, and FISO were generated for each subject and then transformed into MNI space using ANTs.

### Voxel-Based Spatial Statistics

All white matter metric calculations were performed in the study-specific template space. First, a global white matter mask was created from the Harvard-Oxford structural atlas (Johnson, 1997; Schacter et al., 1997; Glisky, 2007; Eckert, 2011). This mask was nonlinearly transformed to each subject’s structural image in template space, to make a subject-specific global white matter mask. All subject-specific masks were then averaged and thresholded at 90% (such that a voxel is accepted into the mask only if the voxel is part of the subject-specific masks for 90% of the subjects). The diffusion metrics were then averaged within this mask to generate global white matter metrics for each subject. The same process was repeated after generating a fornix mask from the JHU White Matter Atlas (Mori, 2007; Wakana et al., 2007; Hua et al., 2008). Note that all statistical analyses in white matter were first performed in the CFE framework. The voxel-based analysis was employed for comparison purposes only.

A global gray matter mask was generated using the Harvard-Oxford structural atlas and metrics were averaged across this mask to calculate global gray matter metrics. The medial temporal lobe was segmented into the CA1, DG/CA3, subiculum, entorhinal cortex, perirhinal cortex, and parahippocampal cortex using an in-house protocol (Kirwan and Stark, 2004; Huffman and Stark, 2014). Diffusion metrics were then averaged across each of these regions of interest in both hemispheres for each subject to make subject-specific bilateral masks. The global gray matter mask was created in the same way as the global white matter mask.

All statistical analyses were performed in Python 3 [using StatsModels (Seabold and Perktold, 2010) or SciPy (Jones et al., 2001)] or GraphPad Prism 8.3.0. (Home—GraphPad, 2019). Statistical *p*-values were corrected for multiple comparisons (six regions of interest for each metric) in all gray matter associated analysis by calculating the false discovery rate (Benjamini et al., 2006). Group differences were computed using student’s two-tailed t-tests in GraphPad Prism 8.3.0. Structural equation analysis was performed using PyProcessMacro (Model 4; PROCESS macro for SPSS and SAS, 2019).

## Results

### Fornix Integrity and Microstructure Are Modulated by Age

**Figure 2:**
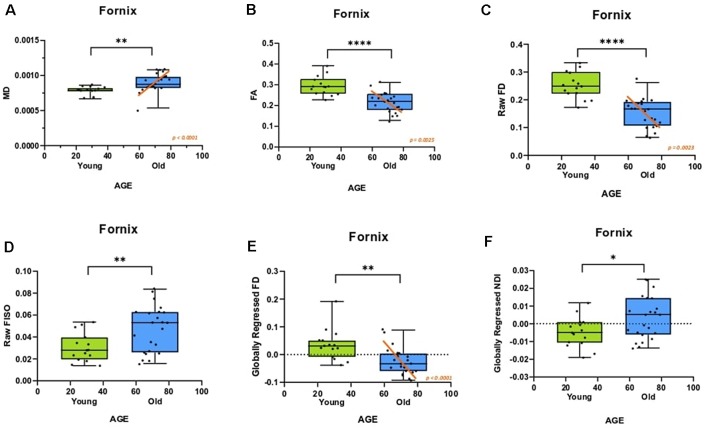
The diffusion metrics of the fornix are influenced by age. Dots indicate individuals with their age both grouped in bars and plotted along the x-axis. **(A,B)** Traditional diffusion tensor metrics of the fornix are linearly correlated with age in the older adults and show group differences. **(C)** The raw fornix fiber density (FD) decreased with age. **(D)** The raw fornix fractional isotropy (FISO) increased in the aged adults. **(E)** The fornix FD maintains its relationship with age even after regressing out global white matter changes. **(F)** The globally regressed fornix neurite density index (NDI) increased with age. Error bars show the standard error of the mean. Asterisks indicate *p* ≤ 0.05, *p* ≤ 0.01, *p* ≤ 0.001, and *p* ≤ 0.0001, respectively.

First, we assessed the effect of age on fornix integrity using the traditional single-tensor diffusion measures of FA and MD. Consistent with prior reports (Bennett et al., 2010; Kantarci et al., 2013), MD in the fornix was reliably higher in the older adults (*t* = 3.118; *p* = 0.0036), while FA showed a significant reduction (*t* = 5.100; *p* < 0.0001). Moreover, both MD and FA were linearly correlated with age in the older adults alone (*R*^2^ = 0.5370, *p* < 0.0001; *R*^2^ = 0.3597, *p* = 0.0025 respectively; Figures 2A,B). We then asked whether similar age-related deterioration in the fornix could be detected with the more sophisticated measures of tract architecture. With CFE statistics, we found that the FD of the fornix was significantly lower in the older adults (*t* = 5.959; *p* < 0.0001) and that FD decreased linearly with age in the older adults alone (*R*^2^ = 0.3638, *p =* 0.0023; Figure 2C). There was no evidence for a relationship between the fiber cross-section of the fornix (*p* = 0.9450) or the raw NDI (*p* = 0.3520) and age. Results also revealed that the amount of free water in the fornix voxels was significantly higher in the older subjects (*t* = 2.773; ρ = 0.0087; Figure 2D). Together, these findings are consistent with the hypothesis that hippocampal connectivity is altered in typical aging and further, that the differences are best attributed to small-scale changes in tract integrity.

To determine whether the differences observed were selective to the fornix and not merely a consequence of age-related global white matter decline, the fornix diffusion metrics were linearly modeled against their respective global white matter diffusion metrics. The residuals of this model were quantified as the “globally regressed” metric. Post global-regression, the FD decline with age remained robust (*t*-test: *t* = 3.4720; ρ = 0.0014; aged-only linear regression: *R*^2^ = 0.6120, ρ < 0.0001; Figure 2E). Interestingly, the globally regressed NDI showed a decrease in the older adults, while this had not been observed with the raw NDI (*t* = 2.277; ρ = 0.0288; Figure 2F), suggesting an age-associated change in the fornix that was not a result of a global decrease. Notably, after removing global effects of age on FISO, there was no remaining effect of age, suggesting that the change observed in the raw fornix FISO was merely a consequence of a global increase in white matter free water concentration. No reliable sex differences were found in any of the fornix metrics after correcting for multiple comparisons. All significant effects were observed in both hemispheres (results reported above were acquired from bilateral masks of a given region of interest).

### Fornix Integrity and Microstructure Correlate With RAVLT Performance

**Figure 3:**
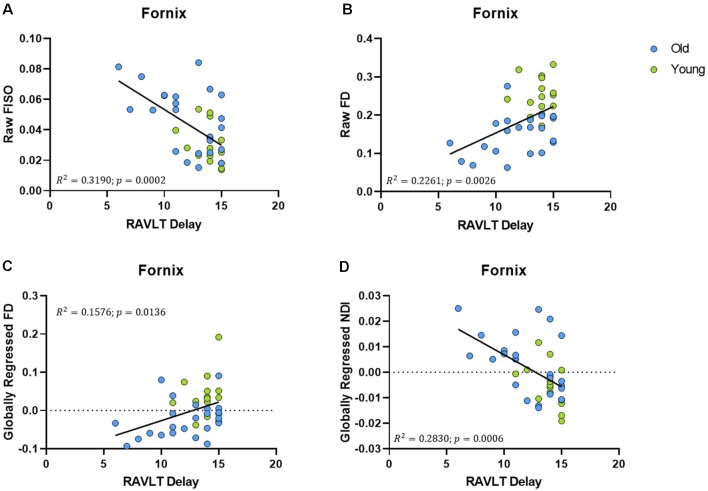
Fornix diffusion metrics were correlated with performance in the rey auditory verbal learning test (RAVLT). **(A,B)** The raw fornix FISO and FD are positively associated with RAVLT performance. **(C,D)** After global regression, the fornix FD and NDI were negatively correlated with RAVLT performance.

We then evaluated whether these individual differences in fornix architecture were correlated with hippocampal-based memory performance. We chose the RAVLT, a word-list learning paradigm, as a standard neuropsychological measure that has proven to be sensitive to age-related changes in memory performance and neural measures (Yassa et al., 2010; Bennett et al., 2015). The raw fornix FISO was negatively linearly correlated with delayed recall RAVLT performance (*R*^2^ = 0.319, *p* = 0.0002; Figure 3A), while the raw FD showed a positive linear relationship with RAVLT performance (*R*^2^ = 0.2261, *p* = 0.0026; Figure 3B). There was no reliable relationship between the raw NDI and the RAVLT score after correcting for multiple comparisons.

After global regression, the FD remained positively correlated with RAVLT delay (*R*^2^ = 0.1576, ρ = 0.0136; Figure 3C). Interestingly, and in conjunction with observations from the previous section, the globally regressed NDI showed a negative linear relationship with RAVLT performance (*R*^2^ = 0.2830, ρ = 0.0006; Figure 3D), suggesting that the age-related decrease in FD and increase in NDI might be contributing to age-associated cognitive decline. The relationship between FISO and RAVLT delay disappeared after global regression, in line with the hypothesis that the observed FISO changes were not unique to the fornix.

### Gray Matter Microstructure of the Medial Temporal Lobe Deteriorates With Age

**Figure 4:**
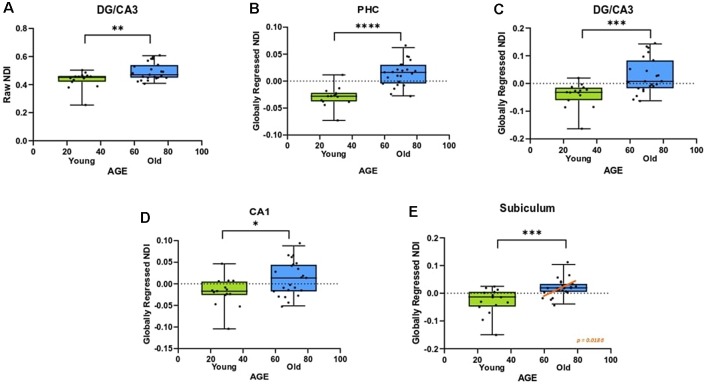
The NDI of the medial temporal lobe is greater in aged adults. **(A)** The raw NDI of the DG/CA3 is increased in aged adults. **(B–E)** After regressing out global gray matter changes, the NDI of the parahippocampal cortices (PHC) and hippocampal subfields are increased in the aged adults. Error bars show the standard error of the mean. Asterisks indicate *p* ≤ 0.05, *p* ≤ 0.01, *p* ≤ 0.001, and *p* ≤ 0.0001, respectively.

An advantage of the NODDI analytic framework is that each voxel is treated as a combination of several different components that lead to the observed diffusion and no distinction is drawn* a priori* as to whether a voxel is gray matter, white matter, or CSF (all voxels are treated as potential mixtures thereof). This approach allows us to perform meaningful analyses of the microstructure of gray matter. In examining whether age induces any structural changes within segments of the medial temporal lobe, we observed that the raw NDI of the DG/CA3 was higher in the aged adults (*t* = 2.863, ρ = 0.0069; Figure 4A). We also observed higher FISO in the perirhinal cortex (*t* = 3.452, ρ = 0.0014), parahippocampal cortex (*t* = 2.913, ρ = 0.0061), DG/CA3 (*t* = 4.667, ρ < 0.0001), CA1 (*t* = 2.897, ρ = 0.0064) and the subiculum (*t* = 5.817, ρ < 0.0001) in older adults. However, we did not observe reliable age-related changes in the diffusion metrics of the entorhinal cortex.

Post-global-regression, there was no age-associated increase in the FISO of the MTL segments, suggesting that the increases observed in the raw metric were simply a consequence of global atrophy due to aging. The globally regressed NDI of the parahippocampal cortex and all hippocampal subfields displayed an age-related increase (PHC: *t* = 5.931, ρ < 0.0001; DG/CA3: *t* = 3.6770, ρ = 0.0008; CA1: *t* = 2.4890, ρ = 0.0176; Subiculum: *t* = 3.8080, ρ = 0.0005; Figures 4B–E). Thus, the NDI change in each of these regions was greater than the global gray matter average NDI in older adults, while it was reduced in the younger adults, suggesting that the NDI increases in the MTL are beyond those associated with age in the whole brain.

No reliable sex differences were found for any of the MTL metrics, after correcting for multiple comparisons. Also, all significant effects were observed in both hemispheres.

### NDI of the DG/CA3 and the CA1 Are Correlated With RAVLT Performance

We next assessed whether the diffusion metrics within the gray matter of the medial temporal lobe were associated with memory ability, particularly performance in the RAVLT. The raw FISO for each medial temporal lobe region, except for the entorhinal cortex, was negatively linearly correlated with RAVLT delay. However, this association disappeared after we regressed out global gray matter metrics, suggesting that the relationship between FISO and RAVLT performance is informed by general global gray matter atrophy driving cognitive decline. Consistent with these results, greater FISO values are thought to be indicative of more necrotic cells and CSF presence in gray matter voxels (Metzler-Baddeley et al., 2012; Ofori et al., 2015).

**Figure 5:**
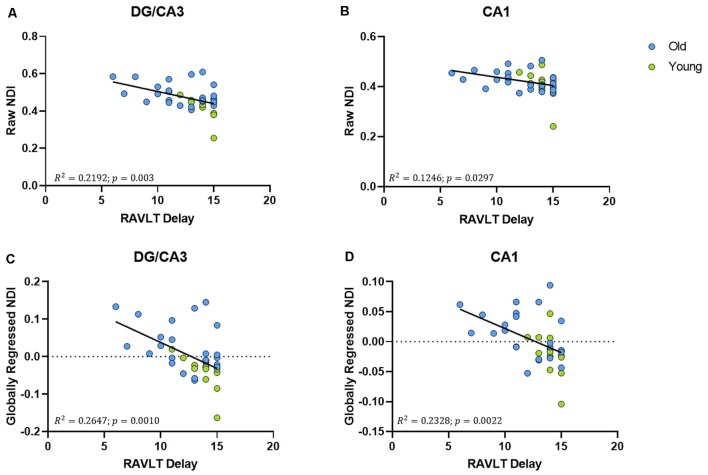
The NDI of the hippocampus has a negative relationship with RAVLT performance. **(A,B)** The raw NDI of the DG/CA3 and CA1 decreased with an increase in RAVLT delay. **(C,D)** This relationship remained after regressing out global gray matter NDI.

The raw NDI of both the DG/CA3 (*R*^2^ = 0.2192, ρ = 0.003; Figure 5A) and the CA1 (*R*^2^ = 0.1246, ρ < 0.0297; Figure 5B) showed a negative correlation with RAVLT performance (however, the effect with CA1 NDI was not reliable after correcting for multiple comparisons). After global regression, the NDI of the DG/CA3 and the CA1 remained negatively correlated with RAVLT performance, even after correcting for multiple comparisons (Figures 5C,D).

### NDI of the DG/CA3, CA1, and the Fornix Remain Correlated With RAVLT Delay, Even After Regressing Age Out

**Figure 6:**
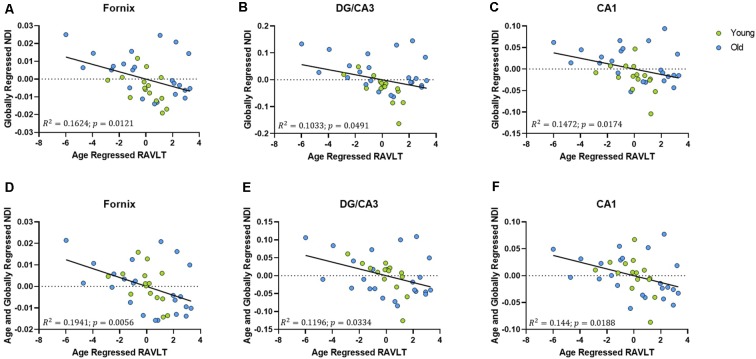
The relationship between RAVLT and NDI is retained even after regressing out the effect of age on both sides, in the fornix, DG/CA3, and CA1. **(A–C)** The globally regressed NDI had a negative relationship with the RAVLT score, after regressing out the effect of age on the RAVLT performance. **(D–F)** This relationship is sustained even after regressing out the effect of age on the globally regressed NDI.

To assess whether these structural correlations with cognitive performance might reflect more than simple age-related decline, we regressed age out of the RAVLT scores in addition to regressing it out of our diffusion metrics. The age-regressed RAVLT scores can be thought of as a “de-aged” RAVLT score (capturing something akin to age-invariant individual differences), as the effects of standard aging are computationally removed from the score. This regressed RAVLT score remained negatively correlated with the globally regressed NDI of the fornix (*R*^2^ = 0.1624, *p* = 0.0121), DG/CA3 (*R*^2^ = 0.1172, *p* = 0.0354), and the CA1 (*R*^2^ = 0.1481, *p* = 0.017). We then regressed age out of the NDI as well and observed that the negative linear correlation survived (Figure 6). The persistence of this relationship between NDI and the RAVLT delay, even after removing the effects of age, suggests that this metric is sensitive to microstructural properties in these regions that directly influence performance in the RAVLT.

**Figure 7:**
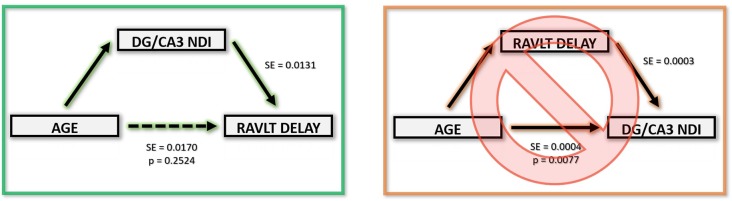
Age-related RAVLT decline can be mediated by an NDI increase in the DG/CA3.

The above correlations suggest a clear relationship amongst age, the integrity of the hippocampus (and its connectivity *via* the fornix), and memory performance. To model the most parsimonious account of these interrelationships, we performed a mediation analysis. An increase in the hippocampus NDI, more specifically, the DG/CA3 NDI, mediated the negative relationship between age and RAVLT delay. Removing the effect of the DG/CA3 NDI change resulted in age having no residual effect on the RAVLT delay. Similarly, a decrease in the FD of the fornix significantly mediated the relationship between age and RAVLT delay. In both cases, we observed that the effect of age on cognitive decline disappeared upon removing the effect of the mediators. No other diffusion metric studied showed this effect (Figure 7). It must be noted that this result must be interpreted cautiously as this is not a longitudinal study and we lack middle-aged participants that would allow for a continuous distribution of ages. However, despite these limitations, this observation bolsters the theory that NDI and FD in DG/CA3 and the fornix respectively may be heavily influencing verbal recall.

### Fiber Cross-Sectional Differences in the Fornix Are Found With Voxel-Based Statistics but Disappear With Fixel-Wise Analyses

It is important to note the differences observed between fixel-based analysis and voxel-based analysis techniques when looking at white matter fiber tracts. A single voxel might have multiple fiber tracts passing through it, causing interference and noise in the measure. Moreover, crossing, fanning and kissing fibers further alleviate this issue as the “density” of a tract may be corrupted by another tract in the voxel that seems to overlap it. This makes most voxel-based analysis techniques undesirably non-specific. A fixel-wise analysis solves this problem by performing statistics on specific fiber populations within the voxel, ensuring that the effect observed is in the pathway that is being studied. This issue is further demonstrated in our observation that connectivity-based fixel-enhancement statistics showed no significant age effects on the fiber cross-section of the fornix, while a voxel-based statistical analysis of the same metric showed a significant difference between age groups (ρ = 0.001). Care must be taken when reporting voxel-based white matter results for this reason. Interestingly, the observed robustness of the fornix fiber cross-section with age suggests that large scale structural changes may not be the focus of age-related architectural changes in the fornix.

## Discussion

In this study, we examined the age-related effects of diffusion metrics of the medial temporal lobe and their relationship with memory performance. We first replicated previous studies showing that the fractional anisotropy of the fornix declines with age, while mean diffusivity increases. We then demonstrated age-related changes in fornix architecture with more comprehensive diffusion metrics, observing a decrease in FD, and an increase in the NDI. Notably, these changes were correlated with poorer RAVLT performance, suggesting that age-related microstructural deterioration of the fornix may play a role in age-related cognitive decline. We also observed similar trends in the gray matter of the medial temporal lobe: showing that the NDI of hippocampal subfields and the parahippocampal cortex increased with age and had a negative correlation with RAVLT performance for DG/CA3 and CA1. Interestingly, we observed that the NDI of the fornix, DG/CA3, and CA1 maintained its relationship with RAVLT performance, even after regressing age out from both the structural metric and the cognitive score, suggesting that this metric is inherently sensitive to a neurobiological property within the hippocampus that corresponds to cognitive performance independent of age. We also demonstrated that traditional methods of analyzing diffusion metrics are not only insufficient for identifying such microarchitectural differences but may also provide unreliable results. Finally, through structural equation modeling, we showed that the DG/CA3 NDI increase and fornix FD decrease mediated age-related decline in RAVLT performance (Table 3).

**Table 3:** Summary of results.

Region	Relationship with age	Relationship with RAVLT
Fornix	FD, GR FD, FISO, GR NDI	FD, GR FD, FISO, GR NDI
DG/CA3	NDI, FISO, GR NDI	NDI, FISO, GR NDI
CA1	FISO, GR NDI	FISO, GR NDI
PHC	FISO, GR NDI	FISO
PRC	FISO	FISO
ERC	-	-
Subiculum	FISO, GR NDI	FISO

*Red: positive relationship. Blue: negative relationship. GR: globally regressed*.

Though previous studies have shown that the integrity of the fornix declines with age, none to our knowledge, have explored this change with more nuanced diffusion metrics to arrive at a more neurobiologically detailed explanation. A decrease in FA could mean a myriad of structural alterations: from demyelination and decrease in axon diameter to a decrease in axon packing density (Sampaio-Baptista and Johansen-Berg, 2017). The fornix has been shown to undergo age-related axonal degeneration in both rats (Naranjo and Greene, 1977) and monkeys (Peters et al., 2010), and our findings on fiber density reductions bolster the idea that an analogous change may be occurring in humans. This, along with our observation that FC remains unchanged, suggests that age-related fornix deterioration may more likely to be caused by microstructural alterations, like changes in axon packing density or loss of myelinated fibers (Peters et al., 2010), more so than macrostructural alterations like an overall reduction in the diameter of the fiber bundle. While this explanation is in no way conclusive, it helps shed more light on the underlying mechanisms behind age-related structural decline. Moreover, the linear relationship we observe between fornix fiber density and age in the older adults alone suggests the existence of a “tipping point” in age- after which fornix deterioration begins to occur consistently and linearly. Unfortunately, the lack of middle-aged adults or longitudinal data prevents us from accurately establishing the age of this tipping point based on these data.

Moreover, NODDI analysis of the diffusion signal enables us to directly compare changes in different tissue types, which is valuable when studying more systemic changes in the medial temporal lobe. As NODDI does not directly discriminate between gray and white matter, we can agnostically measure structural changes and their relationship with cognition. A major caveat of this technique, however, is that there exists very little information on what these metrics may cytoarchitecturally mean. NODDI is a recent technique and its metrics have not been adequately histologically validated, especially in human tissue. The nomenclature of these metrics can also be quite misleading. An increase in the NDI does not necessarily correspond to an actual increase in the number of neurites in a voxel. It must be kept in mind that diffusion-weighted imaging currently does not have the resolution to measure differences at such a microscopic level. That said, NODDI has proven to be extremely valuable in parsing out information from highly complex voxels, and studies that have correlated its metrics with neurobiological properties have been promising (Sepehrband et al., 2015; Sato et al., 2017; Schilling et al., 2018).

With NODDI, we found an increase in FISO with age in the fornix, parahippocampal cortex, perirhinal cortex, and all hippocampal subfields, suggesting that these regions are either getting corrupted by an influx of cerebrospinal fluid or other factors that result in an increase in free water concentration (such as an increase in the number of necrotic cells). Increases in FISO could also be caused by neuropathological factors like edema (Pasternak et al., 2009), inflammation (Wang et al., 2011), and atrophy (Metzler-Baddeley et al., 2011). These factors may also clarify the negative relationship we observed between FISO and RAVLT performance. Interestingly, the effect of age on FISO and the influence of FISO on RAVLT performance disappeared when we regressed out the global change in FISO, suggesting that the increase we had previously observed was merely a consequence of overall brain atrophy with age. More importantly, this global regression introduced an effect of age on the NDI in the fornix, parahippocampal cortex, and all hippocampal subfields, indicating that the NDI in these regions may have a more focused increase than the generalized global metric during aging. This selective increase in NDI may indicate a decrease in dendritic complexity, perhaps caused by atrophy of the surrounding cortical layers (Colgan et al., 2016). This speculation is further invigorated by the retention of the relationship between NDI and RAVLT performance in the fornix, DG/CA3 and CA1, even after regressing out the effect of age in both the structural metric and the cognitive score. A similar dynamic is observed between the fiber density of the fornix and RAVLT performance as well- suggesting that both NODDI and MRtrix may be capable of capturing specialized distortions like reductions in myelination or dendritic complexity. This possibility is especially exciting as its clinical applications are endless: the diagnosis of many neurodegenerative disorders could be aided by the context of the NODDI metrics (Sampaio-Baptista and Johansen-Berg, 2017), with the added advantage that NODDI is relatively easy to implement and process. Another speculation that could rise from the relationship between NDI and RAVLT in the DG/CA3, CA1 and fornix, despite the regression of age, is that this metric is capturing a neurobiological property (like dendritic arborization) that is inherently correlated with verbal recall- suggesting that exploring these metrics might enable us to get at the neurobiological basis of specific cognitive functions.

These findings also raise the speculation that changes in the NDI due to age might be partially driving age-related cognitive decline, at least in the context of delayed verbal recall. This theory is further bolstered with results from our mediation analysis, where we showed that the effect of age on RAVLT performance is no longer reliable once we regress out NDI and FD changes of the DG/CA3 and fornix. Though further evidence is required, these results may indicate that neurite density related structural changes in the fornix and the hippocampus may be responsible for instigating age-related memory decline.

Our results also challenge the validity of current voxel-based analysis methods used in diffusion-weighted imaging, especially in white matter regions. We show that the fiber cross-section of the fornix does not significantly change with age when we performed CFE statistics, but the same measure was sensitive when we computed simple voxel averages about the region of interest. This discrepancy might be explained by the fact that MRI voxels are relatively large and contain multiple fiber pathways running through them, a nuance that simple voxel-based analysis methods do not fully appreciate. For example, the fornix also has other white matter pathways running across it (ex: the cingulum), which may also be changing with age. Fixel-based analysis works around this issue by separating the multiple fiber populations in a single voxel using constrained spherical deconvolution, enabling us to examine individual pathways with more accuracy. More interesting patterns may be found if we map the distributions of metrics across all voxels in the region instead of simply averaging metrics across a region of interest. More specific, non-linear analyses of the voxel-wise distribution of a metric within a given ROI can provide a more comprehensive assessment of how these metrics change. Thus, our results have shown that diffusion-weighted imaging may have more power if we move beyond simple voxel-based averaging analysis methods.

MRtrix and NODDI put together may be able to give us the most detailed view of the microstructure of the live human brain possible with the current technological state of diffusion-weighted imaging. No other study to our knowledge has examined healthy aging-related microstructural changes in the medial temporal lobe and its relationship with cognitive performance at this level of detail before. It is worth noting that our sample size is relatively small and longitudinal data is required to fully determine the progression of these changes. Lack of reverse phase-encoded acquisitions also makes our signal more susceptible to EPI distortions. Moreover, NODDI assumes that intrinsic diffusivity is uniform throughout the brain, but this measure might be susceptible to age-related changes. While these NODDI metrics may be more sensitive to gray and white matter integrity, more research is necessary to understand the underlying neurobiological of each of them. Therefore, future studies tying together diffusion-weighted imaging with the underlying histology will hence be immensely valuable.

## Data Availability Statement

The datasets generated for this study are available on request to the corresponding author.

## Ethics Statement

The studies involving human participants were reviewed and approved by University of California, Irvine Institutional Review Board. The patients/participants provided their written informed consent to participate in this study.

## Author Contributions

SS and CS designed the research. SS collected the data. HR analyzed the data. CS, SS, and HR interpreted the data and wrote the manuscript.

## Conflict of Interest

The authors declare that the research was conducted in the absence of any commercial or financial relationships that could be construed as a potential conflict of interest.

## Footnotes

^1^http://www.mrtrix.org
